# Adding noise to the institution: an experimental welfare investigation of the contribution-based grouping mechanism

**DOI:** 10.1007/s00355-017-1081-5

**Published:** 2017-09-12

**Authors:** Heinrich H. Nax, Stefano Balietti, Ryan O. Murphy, Dirk Helbing

**Affiliations:** 1 Computational Social Science, ETH Zürich, Zurich, Switzerland; 2 Network Science Institute, Northeastern University, Boston, MA USA; 3 Harvard Institute for Quantitative Social Science (IQSS), Cambridge, MA USA; 4 D’Amore McKim School of Business, Northeastern University, Boston, MA USA; 5 Department of Economics, University of Zurich, Zurich, Switzerland

## Abstract

Real-world institutions dealing with social dilemma situations are based on mechanisms that are rarely implemented without flaw. Usually real-world mechanisms are noisy and imprecise, that is, which we call ‘fuzzy’. We therefore conducted a novel type of voluntary contributions experiment where we test a mechanism by varying its fuzziness. We focus on a range of fuzzy mechanisms we call ‘meritocratic matching’. These mechanisms generalize the mechanism of ‘contribution-based competitive grouping’, and their basic function is to group players based on their contribution choices—i.e. high contributors with high contributors, and low contributors with low contributors. Theory predicts the following efficiency-equality tradeoff as a function of the mechanism’s inherent fuzziness: high levels of fuzziness should lead to maximal inefficiency, but perfect equality; decreasing fuzziness is predicted to improve efficiency, but at the cost of growing inequality. The main finding of our experimental investigation is that, contrary to tradeoff predictions, less fuzziness increases both efficiency and equality. In fact, these unambiguous welfare gains are partially realized already at levels where the mechanism is too fuzzy for any high-efficiency outcome to even be a Nash equilibrium.

## Introduction

We conducted an experiment to investigate the welfare consequences of implementing a ‘fuzzy’—rather than a fully precise—mechanism in the context of voluntary contributions games. Our aim is to take a first step in the direction of improving our understanding of the consequences of implementing institutions based on inherently imprecise mechanisms.^1^ This inquiry is relevant before any potential policy recommendations can be made, because real-world implementations would rarely be without flaw, particularly in the context of private, voluntary contributions.

Most investigations of mechanisms in the context of social dilemmas presume that there is perfect observability. Inherently, however, introducing a mechanism in the real world will produce substantial fuzziness. This is due to the fact that players’ individual actions are often not perfectly observable, to the players and to the authority. ‘Imperfect public monitoring’ is ubiquitous in the real world (Abreu et al. 1990; Fudenberg et al. 1994), and there exists a rich body of theoretical investigations in various social dilemma contexts closely related to ours such as noisy prisoners’ dilemmas (Wu and Axelrod 1995) and team production without individual feedback (Alchian and Demsetz 1972).^2^ Previous experimental studies of social dilemma games with imperfect monitoring have revealed that noise may play a crucial, non-trivial role in determining the performance of a mechanism and in sustaining cooperation generally.^3^

In this paper, we investigate a fuzzy mechanism for voluntary contributions games. Voluntary contributions games, as introduced by Marwell and Ames (1979, 1980), provide parsimonious models to capture the strategic interaction underlying public goods provisioning.^4^ In the baseline implementation of these games, individual players make private, costly contributions that create a public good which is then shared equally amongst all players. In the absence of suitable mechanisms, there are insufficient private incentives for contributing behaviors, and universal non-contribution is the unique Nash equilibrium. This is reflected in many economic experiments on voluntary contributions games by a decay of contributions over time (Ledyard 1995; Chaudhuri 2011).

Outcomes with high contributions can only be expected when a suitable mechanism is implemented. Several mechanisms are known.^5^ Successful mechanisms are able to change the incentive structure of the game in such a way that high contributions are stabilized. Numerous lab experiments have shown how voluntary contributions are stabilized through their introduction (Ledyard 1995; Chaudhuri 2011). An important difference between the typical lab setting and the real world is that a real-world implementation of the mechanism would rarely be without flaw. Instead, the real-world mechanism would be fuzzy, that is, subject to various sources of monitoring imperfections, mechanism imprecision and other environmental noise due to, for example, measurement error or enforcement issues.

In this paper, we aim to advance in the direction of understanding fuzzy mechanism implementation. We therefore conduct a novel type of voluntary contributions experiment where we test a mechanism by varying its degree of fuzziness. Our baseline is the mechanism of ‘contribution-based competitive grouping’, as was introduced in a recent, seminal paper by Gunnthorsdottir (2010). Under this mechanism, players are grouped based on their individual voluntary contributions.^6^ As a result, non-contribution is no longer a dominant strategy. Instead, players have incentives to contribute positive amounts if others do likewise in order to be matched with them. Contribution-based grouping changes the game’s entire incentive structure without requiring payoff transfers. Universal non-contribution, the least efficient outcome, continues to be a Nash equilibrium. However, new, more efficient equilibria may also emerge that feature high contribution levels. These new equilibria are characterized by an asymmetric strategy profile such that a vast majority of players contributes fully and a small minority of player free-rides.^7^ Several recent lab experiments confirm coordination on the asymmetric high-efficiency equilibria with high aggregate precision (Gunnthorsdottir 2010; Gunnthorsdottir and Thorsteinsson 2011; Gunnthorsdottir et al. 2010; Rud and Rabanal 2015).

The focus of this paper is on (i) the performance of ‘fuzzy’ variants of Gunnthorsdottir (2010)’s mechanism, and (ii) on their welfare consequences in terms of efficiency and equality. Fuzzy variants of Gunnthorsdottir (2010)’s mechanism were recently formulated by Nax et al. (2014) introduction of an additional parameter (interpretable as variance) that measures the *degree of imprecision* inherent to the competitive grouping’s basic functioning.^8^ This fuzzy generalization of contribution-based group matching is referred to as ‘meritocratic matching’. Compared to the basic mechanism, meritocratic matching works as follows: instead of grouping players based on actual contributions, actual contributions are ‘noised’ by variance $\sigma ^2$ (measuring fuzziness/imprecision), and players are grouped based on these noised contributions. Meritocratic matching thus bridges the no-mechanism case of random matching and contribution-based competitive grouping continuously: when $\sigma ^2=0$ the mechanism corresponds to contribution-based competitive grouping; when $\sigma ^2\rightarrow \infty$ the mechanism approaches random re-matching as in a standard implementation (Andreoni 1988). Nax et al. (2014) show that high-efficiency equilibria exist provided the mechanism is precise enough implying a bound on $\sigma ^2$.

In this study, we investigate the welfare properties of meritocratic matching for a wide range of $\sigma ^2$ values. Theory predicts existence of high-efficiency Nash equilibria for some of these values, but not for all, and not for $\sigma ^2\rightarrow \infty$. Our results summarize as follows. We confirm that the asymmetric high-efficiency equilibria are coordinated upon with high aggregate precision when they exist, validating theory predictions and previous lab studies. Contrary to theoretical predictions, however, we provide novel evidence that higher levels of meritocracy also increase ex post equality. Moreover, we find that these unambiguous welfare gains are even realized when the mechanism is too fuzzy for theory to even predict existence of high-efficiency equilibria.

We view high fuzziness as the realistic ‘default’ implementation of our mechanism. However, a policy maker could somehow choose to reduce the mechanism’s inherent noise (e.g. by investing into monitoring or implementation technologies). Viewing the mechanism’s inherent noise, therefore, as some sort of policy variable and without explicitly modeling the involved costs of ‘setting the noise’, we can draw the following main policy conclusion from our results: provided that there is ex ante equity (as there was in our experiment by design), it would be unambiguously welfare-beneficial to introduce meritocratic matching, even if the mechanism remains very fuzzy.

The remainder of this document is structured as follows. Next, we provide details of the model and of the experimental design. In Sect. 3, we present the results of our welfare investigation. Finally, we conclude in Sect. 4.

## The experiment

### Modified voluntary contributions games

A fixed population of *n* agents, $N=\{1,2,\ldots ,n\}$, plays the following *modified voluntary contributions game* repeatedly through periods $T=\{1,2,\ldots ,t\}$.

1. **Contributions.** Each agent *i* simultaneously decides to *contribute* any number of coins $c_i$ between zero and his budget $B>0$. The amount $B-c_i$ not contributed goes straight to his/her *private account*. The ensemble of players’ decisions is represented by the vector of actual contributions *c*.
2. **Fuzz.** Fuzz in the form of i.i.d. Gaussian noise with mean zero and variance $\sigma ^2\ge 0$ is added to each actual contribution $c_i$ which results in the noised contribution vector $c'$.
3. **Grouping.** *k* groups of a fixed size $s<n$ (such that $s*k=n$) are formed according to the ranking of the noised contributions $c'$ (with random tie-breaking).^9^ That is, the highest *s* contributors according to $c'$ form group $G_1$, the next highest *s* contributors form $G_2$, etc. The resulting group partition is $\rho =\{G_1,G_2,\ldots ,G_k\}$.
4. **Payoffs.** Finally, based on grouping and the actual (not noised!) contributions vector *c*, payoffs $\phi$ realize as follows. Each player *i*, matched into $G_i$ with $j\ne i$, receives the standard linear public goods payoff of:
  1 $$\begin{aligned} \underbrace{\phi _i(c)}_{\text {payoff}}= \underbrace{(B-(1-m)*c_i)}_{\text {return from private account}} + \underbrace{\sum _{j\in G_{-i}} m * c_j,}_{\text {return from group account}} \end{aligned}$$
  where *m* represents the marginal per capita rate of return, and $G_{-i}$ indicates the members of group $G_i$ excluding *i*.

*Aside*. Note that we will think of high levels of the variance $\sigma ^2$ as the ‘default’ setting for implementing contribution-based grouping. However, we consider the possibility that the policy maker can make investments to decrease $\sigma ^2$ and to make the mechanism more precise, and therefore think of $\sigma ^2$ as a sort of policy variable in our subsequent analysis. Recall that the cases $\sigma ^2=0$ coincide with Gunnthorsdottir (2010)’s game (here, ‘perfect meritocracy’), and $\sigma ^2\rightarrow \infty$ with random re-matching (here, ‘no meritocracy’). Associating setting $\sigma ^2$ with a ‘cost’ is left as an avenue for future research; here we consider the case when these costs are negligible compared with the benefits at stake.

#### Parameter choices

In our design, we consider games where 4 groups of size 4 form ($n=16$ and $k=4$) with a budget of $B=20$ each period and a marginal per capita rate of return $m=0.5$. The choice of parameters ensures good comparability of results with, on the one hand, the literature on voluntary contributions games under random re-matching (as reviewed by Ledyard 1995; Chaudhuri 2011), and, on the other hand, with contribution-based competitive grouping mechanisms. Our treatments vary with respect to values and orders of values of $\sigma ^2$, that is, which mechanisms are being played and in which order.

Before performing the main experiment, we tested online on Amazon Mechanical Turk (AMT) the following $\sigma ^2$-values: 0, 2, 4, 5, 10, 20, 50, 100, 1000, and $\infty$. This way, we could quickly explore the behavior of the participants in the vast parameter space. Previous research has suggested that the data quality of AMT experiments is adequate and reliable in various settings (Wang et al. 2015; Hauser and Schwarz 2016; Arechar et al. 2017). In fact, in our study, the behavior of participants in our online sessions did not significantly deviate from the known patterns found in the literature, or from the behavior of participants in the closest corresponding laboratory sessions from our own study except for the $\sigma ^2=\infty$ condition.^10^

Using insights from the online trials and from computer simulations (Nax et al. 2014), we then chose four values of $\sigma ^2$ for the main laboratory experiment: 0, 3, 20, and $\infty$. The $\sigma ^2$-values of 0 and $\infty$ were chosen as they represent the perfect mechanism implementation by Gunnthorsdottir (2010) and random group matching, respectively. The $\sigma ^2$-values of 3 and 20 were chosen mainly because the simulations by Nax et al. (2014) indicated interesting ‘tipping’ properties at 3 and 20.^11^ Furthermore, 3 and 20 are safely within the variance interval where both ’high’ and ’zero’ equilibria exist. In fact, all the sigma values can be grouped together in different regimes based on interpretation and equilibrium analysis, as shown in Table 1.

**Table 1:** Meritocracy regimes with corresponding variance intervals and equilibrium structure

Merit. regime	Variance	Equilibria	Experiment
PERFECT	$\sigma ^2=0$	‘high’ and ‘zero’	LAB $(\sigma ^2=0,n=48)$
HIGH	$\sigma ^2=(0,20)$	‘high’ and ‘zero’	LAB $(\sigma ^2=3,n=48)$
LOW	$\sigma ^2=[20,75)$	‘high’ and ‘zero’	LAB $(\sigma ^2=20,n=48)$
NO (ZERO)	$\sigma ^2=\infty$	Uniquely ‘zero’	LAB $(\sigma ^2=\infty ,n=48)$
INSUFFICIENT	$\sigma ^2=[75,\infty )$	Uniquely ‘zero’	AMT $(\sigma ^2=[100,1000],n=59)$

Last column reports the source for the experimental data, the number of participants and the actual variance levels used in the analysis

Throughout the paper we will focus on the results from the laboratory experiments. We will use the results from online sessions with $\sigma ^2$-values of 100 and 1000 pooled together to complement the analysis to a region of the parameter space not covered by our laboratory sessions.

#### Nash equilibrium play: existence and intuition

As characterized in Gunnthorsdottir (2010) and Nax et al. (2014), there exist two types of Nash equilibria. One equilibrium is such that all players contribute zero. This equilibrium always exists, for all specifications of the game and for all variance levels. It coincides with the outcome that generates the lowest total payoffs. Another outcome that is a candidate for Nash equilibrium is characterized by full contribution by a vast majority of players and by free-riding of a few. This outcome produces close to the maximum of possible total payoffs, and it exists for certain parameter and noise specifications, in particular when the marginal per capita rate of return is high enough and when the noise is not too large.

To get some intuition for these Nash equilibria one has to evaluate the expected payoff, $\mathbf {E}\left[ \phi _i(c)\right]$, that the players foresee during the decision stage, i.e. before groups are formed. In Eq. (1), the first term on the right-hand side, i.e. the private-account return, is completely determined by the agent’s own contribution choice. There is no uncertainty. The second term on the right-hand side—i.e. the group-account return—however, may depend on the player’s own and on others’ contributions in a probabilistic way.

*Special case of NO-MERIT*. Only in the case of zero meritocracy (i.e. random re-matching with $\sigma ^2=\infty$), grouping is completely independent of the contribution decisions. Hence, $\mathbf {E}\left[ \phi _i(c)\right]$ behaves as it does in the standard voluntary contributions game: payoff is strictly decreasing in each player’s own contribution. Hence, non-contribution is a strictly dominant strategy, and the only equilibrium is universal non-contribution (indicated by ‘zero’ in Table 1).

*General case*. For meritocratic matching with any finite $\sigma ^2\ge 0$, the player’s contribution decision affects the probability of being matched into different groups. Deciding to make a positive/higher contribution comes with a tradeoff between the sure loss on the own contribution (private account) and the promise of a higher return from being matched with others’ contributions (group account). Non-contribution is no longer a strictly dominant strategy, provided the promise of a higher group return is likely enough, that is, if the variance is not too large. Crucially, the probability to be matched accurately according to one’s actual contribution is decreasing as $\sigma ^2$ increases, and noised contributions become less and less accurate representations of actual contributions.

Nax et al. (2014) show that—and this generalizes the results by Gunnthorsdottir (2010)—if the level of meritocracy is sufficiently large in addition to a bound on *m*, there exist high-efficiency pure-strategy Nash equilibria where a large majority of players contributes the full budget *B* and a small minority of players contributes nothing (This equilibrium is indicated by ‘high’ in Table 1). The intuition behind that kind of equilibrium is that those who contribute do so in the hope of being matched with others doing likewise (which happens with a high probability), while the free-riders expect to be able to free-ride on the unlucky contributors who end up being matched with them.

The outcome where all players contribute zero, indicated by ‘zero’ in Table 1, continues to be an equilibrium for any level of $\sigma ^2$ too.^12^ This is because no players can individually benefit from the grouping mechanism, no matter how precise it is, if he/she is the only player contributing a positive amount.

*Equilibria for our parameters.* For our parameter choices ($n=16$, $k=4$, $B=20$ and $m=0.5$), Nash equilibrium existence is summarized in Table 1: for treatments with $\sigma ^2\in \{100, 1000, \infty \}$, the only equilibrium is ‘zero’; for treatments with $\sigma ^2\in \{0, 2, 3, 4, 5, 10, 20, 50\}$, one equilibrium is ‘zero’ and in addition there are ${n\atopwithdelims ()2}$ asymmetric pure-strategy ‘high’ equilibria where exactly 2 players free-ride and 14 others contribute fully.^13^

### Experimental details

Altogether we ran 28 experimental sessions with a total of 434 participants using the new experimental software NodeGame (Balietti 2016). We shall now briefly summarize our experimental design. More details about the experiment can be found in Appendix A.2.

12 Experimental sessions with a total of 192 participants were run at the ETH Zürich Decision Science Laboratory (DeSciL). Each lab session lasted roughly one hour. There were 16 participants in each session. DeSciL recruited the subjects from the joint subject pool of the University of Zurich and ETH Zurich maintained by the University Registration Center for Study Participants (UAST). The experiment followed all standard behavioral economics procedures and meets all Ethics Committee guidelines. Decisions, earnings and payments were anonymous. Payments were administered by the DeSciL’s lab staff. In addition to a 10 CHF show-up fee, each subject was paid according to a known exchange rate of 0.01 CHF per coin. Overall, monetary rewards ranged from 30 to 50 CHF, with a mean of 39 CHF. Each lab session consisted of two games, each of which was a 40-round repetition of the same underlying stage game.^14^ The same fixed budget was given to each subject every period. Each game had separate instructions that were distributed at the beginning of each game. These instructions contained full information about the structure of the game and about the payoff consequences to themselves and to the other agents. After reading the instructions, all participants were quizzed to make sure they understood the task. The two games had different variance levels. There were four variance levels in our lab study, $\sigma ^2=\{0,3,20,\infty \}$, and each game had equivalent instructions. We played every possible pair of variance levels in both orders to have an orthogonal balanced design. As the game went on, players learned about the other players’ previous actions and about the groups that formed.

The remaining 16 experimental sessions were run on Amazon’s Mechanical Turk (AMT) with a total of 242 participants. In each AMT session, all participants played only one game with one of the following variance levels from $\sigma ^2=\{0,2,4,5,10,20,50,100,1000,\infty \}$. In order to mitigate dropout problems, AMT sessions were shorter with only 20 or 25 rounds. AMT sessions lasted 20 min on average, and subjects earned between 1.7 and 3.4 USD.

## Results

**Fig. 1:**
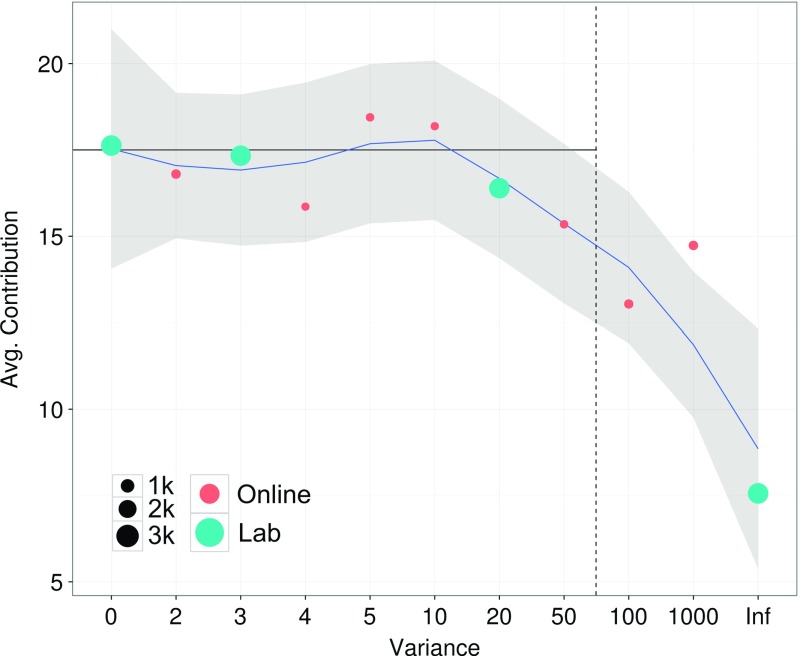
Average contributions for various variance levels (lab and AMT). Sizes of dots are proportional to the number of observations. The black horizontal line shows the contribution level of the near-efficient equilibria, and the dashed vertical line highlights the noise level beyond which the zero-contribution equilibrium becomes the unique equilibrium

Figure 1 summarizes the average contributions. These paint a coherent picture of how, as expected, contributions tend to decrease as the mechanism becomes fuzzier, with only one exception in the range of ‘insufficiently’ meritocratic matching treatments. This is true for both laboratory and online sessions.

**Fig. 2:**
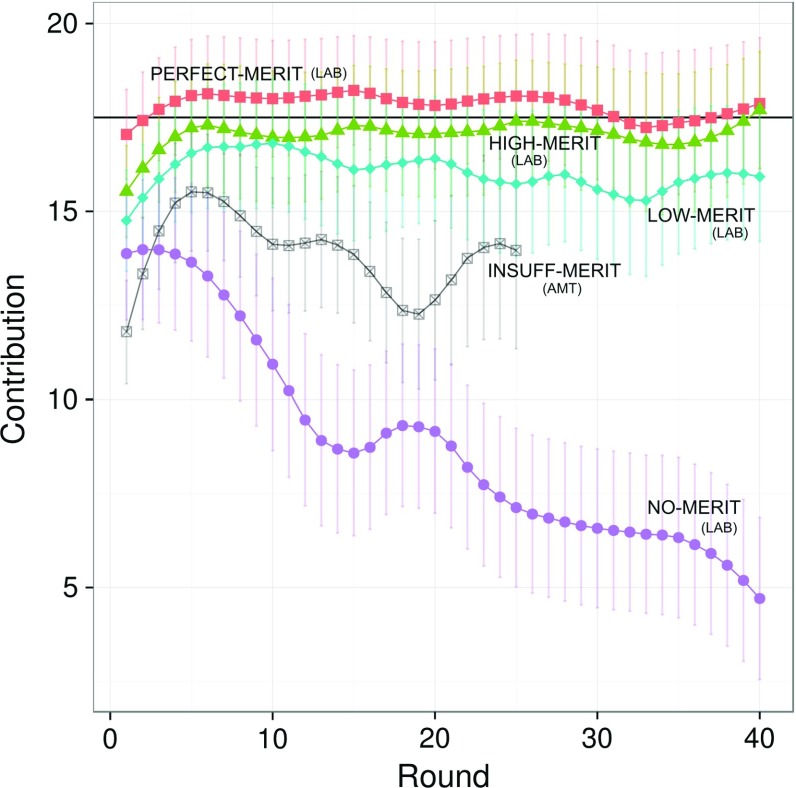
Contributions over time for PERFECT-, HIGH-, LOW-, INSUFF- and NO-MERIT, respectively, associated with $\sigma ^2=\{0,3,20,\{100,1000\},\infty \}$. Error bars show 95%-confidence intervals. PERFECT-, HIGH-, and LOW-MERIT (from lab sessions) are stable compared to the decay of NO-MERIT. Data for INSUFF-MERIT (from AMT sessions) shows intermediate patterns. The black horizontal line shows the near-efficient equilibrium

Figure 2 shows the average contribution levels over rounds. Conditions LOW, HIGH and PERFECT-MERIT sustain high levels of contributions, close to the high-efficiency equilibrium. For the case of NO-MERIT, the steadily declining contributions reflect the usual pattern (Ledyard 1995; Chaudhuri 2011). For INSUFFICIENT-MERIT, unfortunately with slightly shorter series from AMT, we observe no such decline, but intermediate contribution levels throughout instead.

Overall, the mean level of contributions among the four lab treatments is significantly different (linear mixed model LMM $F_{3,8} = 36.8, P < 0.0001$).

Our main analysis focuses on the laboratory data, that is, on conditions PERFECT, HIGH, LOW and NO-MERIT with $\sigma ^2=\{0,3,20,\infty \}$. First, we study the efficiency, inequality and fairness properties of the data by analysis of the first game played in each session. Subsequently, we analyze data from the second game to assess the participants’ sensitivity to changes in meritocracy levels.

### Efficiency

**Fig. 3:**
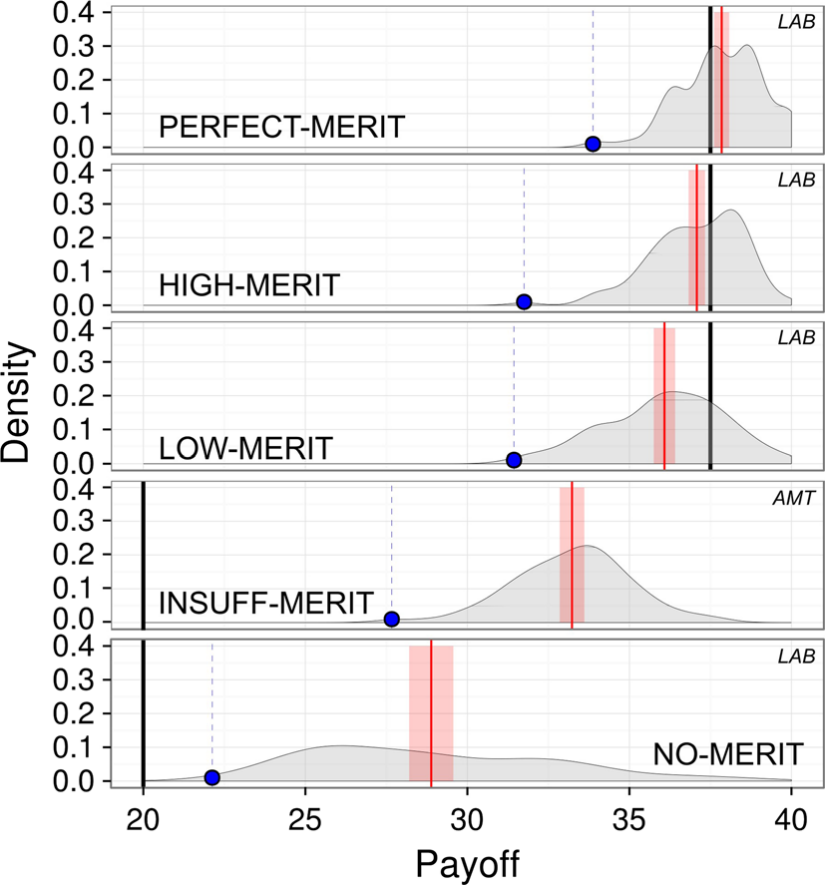
Payoff efficiency for PERFECT-, HIGH-, LOW-, INSUFF- and NO-MERIT, respectively, associated with $\sigma ^2=\{0,3,20,\{100,1000\},\infty \}$. Efficiency increases as meritocracy increases. Black solid lines indicate the mean payoff as implied by the ‘high’ equilibria, red solid lines indicate the mean payoff observed in the experiment, red-shaded areas indicate the 95%-confidence intervals of the mean. Blue dots indicate the payoff of the worst-off player (note that the worst-off player in any equilibrium receives twenty ‘coins’)

By ‘efficiency’ we refer to average total payoffs, $\overline{\phi }=\frac{\sum _{i\in N}\phi _i}{n}$, over the forty rounds, which are linearly related to total contribution levels. Theory predicts existence of high-efficiency equilibria for LOW-, HIGH- and PERFECT-MERIT, but not for INSUFFICIENT- and NO-MERIT. We shall show that play coordinates on these high equilibria where they exist, which replicates previous experimental results for PERFECT-MERIT (Gunnthorsdottir 2010; Gunnthorsdottir and Thorsteinsson 2011; Gunnthorsdottir et al. 2010). Indeed, the levels of efficiency supported by the ‘high’ equilibria under LOW, HIGH and PERFECT-MERIT approximate well theoretical predictions, while the inefficiency prediction of the ‘zero’ equilibrium under no-meritocracy (INSUFFICIENT and NO-MERIT) largely understates the achieved efficiency levels. Figure 3 summarizes this analysis.

Overall, we observe significant differences in the mean of realized payoffs among the four lab treatments (linear mixed model LMM: $F_{3,8} = 36.95, P < 0.0001$). Taking NO-MERIT as a baseline, LOW-MERIT led to an increase in the average realized payoff of 7.1611 (Likelihood Ratio Test LRT: $\chi _{(1)} = 12.7, P = 0.0004$), HIGH-MERIT to an increase of 8.1964 (LRT: $\chi _{(1)} = 17.48, P < 0.0001$), and PERFECT-MERIT to an increase of 8.8287 (LRT: $\chi _{(1)} = 16.22, P < 0.0001$). These levels correspond to roughly double those of NO-MERIT. Computing the most conservative (Bonferroni) adjusted *p*-values on all pair-wise differences reveals that the treatment with variance $\infty$ is significantly different ($P < 0.0001$) from the other three variance levels $\sigma ^2=\{0,3,20\}$, which are themselves not significantly different from each other.

For intermediate meritocracy regimes $\sigma ^2=\{3,20\}$, efficiency is significantly below the level implied by the payoff-dominant equilibria,^15^ but the difference is small (two and eight percent respectively). Conversely, under full meritocracy $\sigma ^2=0$, efficiency is above and within two percent of equilibrium.

### Equity by design versus ex ante/ex post inequality

Note that our experimental design features ex ante ‘equity’ in the sense that the games we study are symmetric in every respect including budgets. Note also that the non-contribution ‘zero’ equilibrium (which exists for all $\sigma ^2$) is also characterized by perfect equality in outcomes, independent of whether evaluated ex ante (at the contribution decision stage) or ex post (after payoffs realize).

By contrast, the near-efficient ‘high’ Nash equilibria (which exist for $\sigma ^2<75$) are asymmetric and predict that 14 out of 16 players contribute fully and 2 players free-ride. This asymmetry implies both ex ante (to a lesser extent) and ex post (to a larger extent) inequality.^16^ Ex post inequality in the high-efficiency equilibria, in particular, is quite serious as the two free-riders who get matched with at least two full-contributors are amongst the best-off players, while some lucky full-contributors are better-off (those not matched with free-riders) than other unlucky full-contributors (those matched with free-riders) who are substantially worse-off.

In this section, we shall show that laboratory evidence yields diametrically opposite results compared with what theory predicts regarding ex post equality; namely, contrary to theoretical predictions, higher meritocracy levels lead to outcomes that are more equal in terms of payoff distributions than lower meritocracy regimes. This is because players play less heterogeneously and more in line with equilibrium in high meritocracy regimes than in lower ones.

One can identify two measures of payoff inequality directly from the moments of the payoff distribution: (i) the payoff of the worst-off (Rawls 1971), $\underline{\phi }=\min \{\phi _i\}$, and (ii) the variance of payoffs, $\sigma ^2=\frac{\sum _{i\in N}(\phi _i-\overline{\phi })^2}{n}$. A more sophisticated third alternative is (iii) the Gini coefficient. In terms of all measures, our analysis shows that equality increases with meritocracy. Note that the following results are also robust to other measures of inequality (Cowell 2011) (see “Appendix”).

**Fig. 4:**
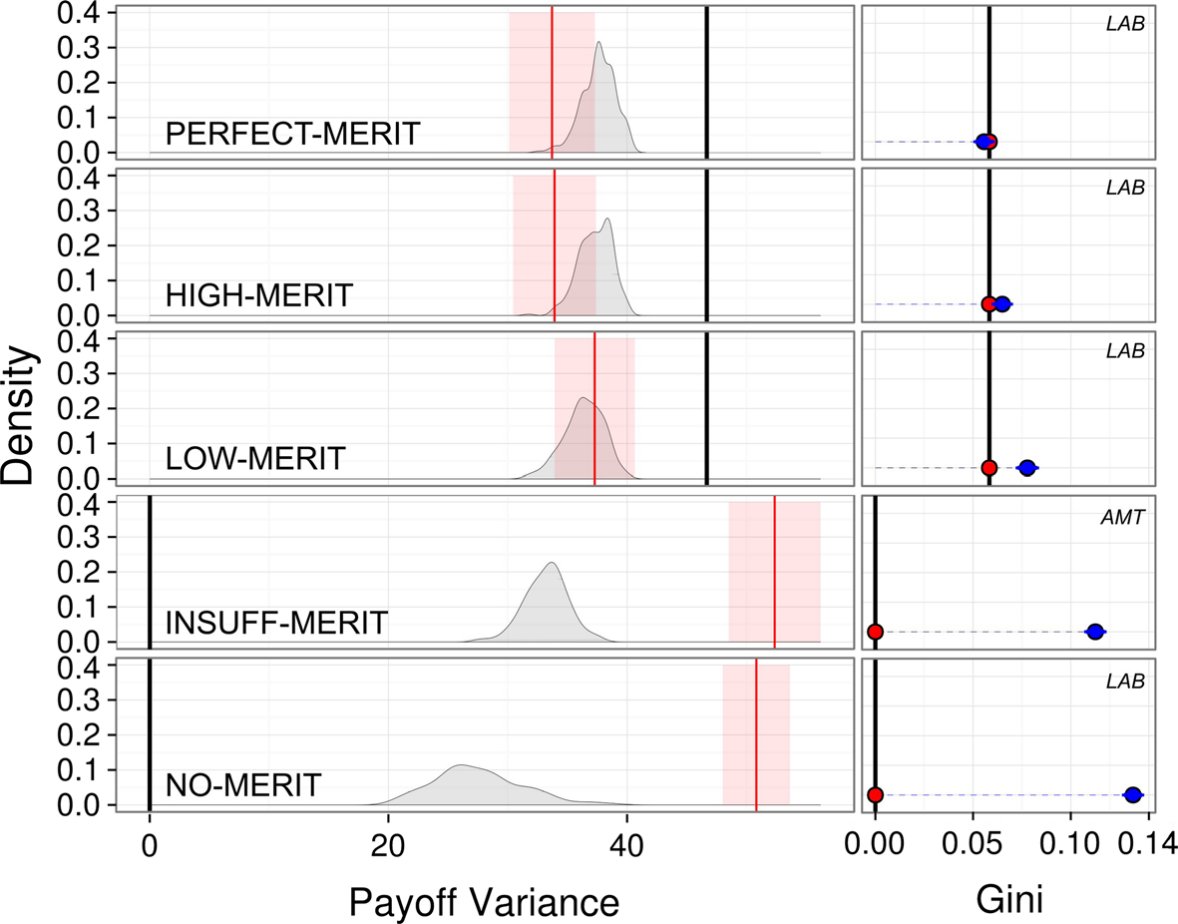
Payoff inequality for PERFECT-, HIGH-, LOW-, INSUFF- and NO-MERIT, respectively, associated with $\sigma ^2=\{0,3,20,\{100,1000\},\infty \}$. Inequality, measured by the average variance of payoffs and by the Gini coefficient, decreases as meritocracy increases. Left panel: Smoothed distributions of average variance over 40 rounds. Black solid lines indicate the variance of the payoffs as given by the ‘high’ equilibria, red solid lines indicate the mean variance observed in the experiment, red-shaded areas indicate the 95%-confidence intervals of the mean variance. Right panel: Average Gini coefficient of the distribution of payoffs with 95%-confidence intervals. Black solid lines and and red dots indicate the Gini coefficient implied by the equilibrium

Figure 4 summarizes our analysis. It highlights that, as with efficiency—but this time contrary to theoretical predictions—equality also increases from $\sigma ^2=\infty$ (NO-MERIT) through $\sigma ^2=\{20,3\}$ to $\sigma ^2=0$ (PERFECT-MERIT). A significant difference in the variance of realized payoffs in each round among the four treatments is found (LMM: $F_{3,8} = 7.27, P < 0.0113$). When computing Bonferroni adjusted *p*-values, the treatment with variance $\infty$ was found significantly different ($P = 0.0003 ; P = 0.0004 ; P = 0.0086$) from the other three variance levels ($\sigma ^2=\{0,3,20\}$), which are themselves not significantly different from each other. Taking NO-MERIT as a baseline, LOW-MERIT led to a decrease in the variance of realized payoffs in each round of −13.546 (LRT $\chi _{(1)} = 8.13, P = 0.0043$), HIGH-MERIT to a decrease of −16.914 (LRT $\chi _{(1)} = 9.89, P = 0.0016$), and PERFECT-MERIT to a decrease of −17.122 (LRT $\chi _{(1)} = 6.78, P = 0.0091$).

These decreases in inequality are also reflected by other inequality measures, in particular by differences in the Gini coefficient and by the order of the payoff of the worst-off (i.e. a Rawlsian equality measure).^17^ In summary, under NO-MERIT, equality is significantly below the level implied by equilibrium. For all three positive levels of meritocracy, equality is above that achieved by NO-MERIT and above the theoretically implied levels. INSUFFICIENT-MERIT features a higher level of variance than NO-MERIT due to the large difference in sample size. However, when looking at the Gini coefficient, its value lies between that of LOW and NO-MERIT.

### Interpretations

We have found that Nash predictions fare well in approximating efficiency levels in the fuzzy regimes LOW- and HIGH-MERIT, and in the perfect implementation PERFECT-MERIT. Nash equilibrium was neither implemented nor converged to in INSUFFICIENT-MERIT. In NO-MERIT, there was aggregate convergence toward equilibrium (decay of contributions). This section is dedicated to different behavioral explanations of these phenomena under the variously fuzzy mechanisms.

#### Fairness in meritocratic matching

In our analysis of Nash equilibria, we considered the theoretic case where all subjects were risk-neutral and pursued an entirely selfish, linear payoff function. From a wide variety of experiments, however, we know that players are risk-averse and pursue distributional and other-regarding preferences that take into account how their decisions affect not only their own material payoff but also the material payoffs of others. In particular, human preferences have been shown to include ‘fairness’ considerations. Amongst the best-known models of preferences for fairness are the models of Fehr and Schmidt (1999), Bolton and Ockenfels (2000) and Charness and Rabin (2002).

Fairness models have been used to explain why subjects in voluntary contributions games, in the absence of a mechanism such as our contribution-based meritocracy, may initially contribute differing positive amounts. As players contribute different amounts under random re-matching, in particular, those contributing more earn less (which is *disadvantageously* unfair), and those contributing less earn more (which is *advantageously* unfair). Numerous experiments have shown that experiences of unfair outcomes lead to contribution adjustments, and that experiences of disadvantageously unfair outcomes, especially, lead to contribution reductions. Therefore, these kinds of fairness considerations, where contribution reductions due to disadvantageously unfair experiences outweigh the corresponding contribution increases due to advantageously unfair experiences, lead to a spiraling down of conditional cooperation (Fischbacher et al. 2001). This is a phenomenon we also observe in our NO-MERIT baseline treatment.

Fairness motives are likely to be different when a mechanism is in place. Consequently, which fairness notions are relevant in evaluating outcomes may depend on the underlying principle of the mechanism. The basic principle of the mechanisms considered in this paper is to group contributors with contributors, and to group free-riders with free-riders. As this mechanism becomes more fuzzy ($\sigma ^2$ increases), this principle is more frequently violated, and free-riders may even be matched into better groups than contributors. Subjects may find this unfair, in light of what the mechanism is designed to achieve in principle, and react to this by adjustments of their behavior. To explore these reactions we propose a notion of fairness in light of our mechanism, which we term ‘meritocratic fairness’. We contrast this notion with the aforementioned ‘payoff fairness’ concerns inherent to models such as Fehr and Schmidt (1999), Bolton and Ockenfels (2000) and Charness and Rabin (2002).

Meritocratic fairness is defined as follows: an outcome is fair in light of our mechanism’s meritocracy basic principle if all players are matched according to their actual contributions—which is what the perfect mechanism with $\sigma =0$ is designed to do. Similarly, an outcome is unfair if there exists at least one player who contributed less (more) than another and is matched into a group with higher (lower) contributions. Formally, *meritocratic unfairness*, in terms of both advantageous ($\textit{MU}_{Adv}$) and disadvantageous ($\textit{MU}_{Dis}$) considerations, is measured by the following two quantities:

2 $$\begin{aligned} \begin{aligned} MU_{Dis}&= \frac{1}{n-s} * {\sum }_{j\in N}\max (\varDelta _{ij},0)*\max (\varDelta _{G_j G_i},0),\\ MU_{Adv}&= \frac{1}{n-s} * {\sum }_{j\in N}\max (\varDelta _{ji},0)*\max (\varDelta _{G_i G_j},0),\\ \end{aligned} \end{aligned}$$

where for any pair of players, *i* and *j*, in groups $G_i$ and $G_j$ ($i \ne j$), $\varDelta _{ij}$ represents the difference in contributions $c_i-c_j$, and $\varDelta _{G_i G_j}$ is the difference in average group contributions $\frac{1}{4}\sum _{k\in G_i}c_k-\frac{1}{4}\sum _{k\in G_j}c_k$.^18^

#### Contribution decisions: meritocratic fairness and strategic concerns

Contributions in our model play a doubly strategic role. On the one hand, they determine a player’s payoff within a given group. On the other hand, they also determine the group into which the player is matched. As regards individual contribution decisions, we conjecture that fairness considerations matter, and that the relevant fairness considerations are adapted to the functioning of our mechanism and inherent noise with which the mechanism is announced. Hence, we test the following two higher-order hypotheses. $(H_1)$ Players will adjust their contributions after experiences of unfair outcomes. $(H_2)$ What is considered unfair will depend on the mechanism that is in place.

As with the standard notion of unfairness (here evaluated according to Fehr and Schmidt 1999), we expect that the consequences of the distaste for meritocratic unfairness are such that a player responds by decreasing (increasing) his/her contribution after experiencing disadvantageous (advantageous) meritocratic unfairness. This represents our testable hypothesis $(H_1)$. Furthermore, we expect—according to $(H_2)$—that meritocratic fairness considerations will matter more in mechanism implementations with less noise, and that standard fairness considerations (here Fehr and Schmidt 1999) will matter more in mechanism implementations with more noise. Our hypotheses lead to the following predictions in our different treatments:

- Under PERFECT-MERIT, starting at the near-efficient Nash equilibrium prediction, we do not expect significant departures from a strategic best-response state as there is no inherent meritocratic unfairness (by definition), and we expect standard fairness considerations to be less important.
- For the intermediate meritocracy levels (HIGH-, LOW-, INSUFFICIENT-MERIT), we expect contribution decreases in response to meritocratic unfairness experiences. This effect is expected to be weaker the higher the noise of the implementation. However, other than under NO-MERIT, downward corrections of contributions would not necessarily need to trigger an overall downward decay of contributions, because of the different strategic incentives. We expect standard fairness considerations to be of limited importance, and of increasing importance as noise increases.
- Under NO-MERIT, we expect meritocratic fairness to play no role, as the mechanism has no such function. Instead, we expect standard fairness considerations to matter, which will lead to downward corrections and to an overall downward decay of contributions.

#### Meritocratic fairness: results

**Fig. 5:**
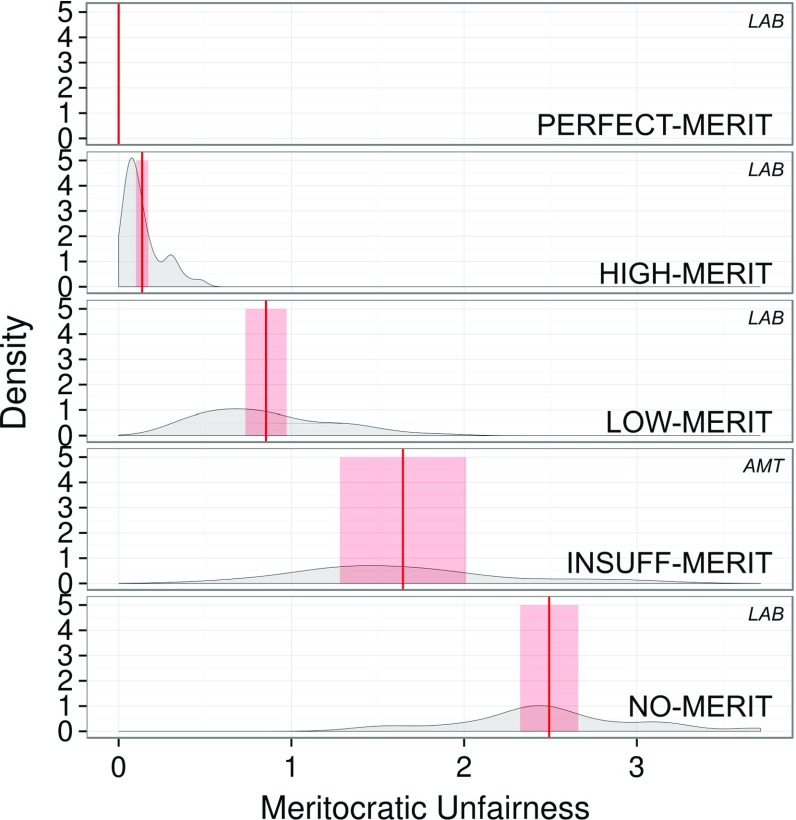
Meritocratic unfairness for PERFECT-, HIGH-, LOW-, INSUFF- and NO-MERIT, respectively, associated with $\sigma ^2=\{0,3,20,\{100,1000\},\infty \}$. Smoothed distribution of average meritocratic unfairness per round. Unfairness decreases as meritocracy increases. Red solid lines indicate the mean level of meritocratic unfairness observed in the experiment, red-shaded areas indicate the 95%-confidence intervals of the mean

Figure 5 shows the distributions of meritocratic unfairness across different treatments. Similarly to efficiency and inequality, we find increases in meritocratic fairness from NO-MERIT through all meritocracy levels up to PERFECT-MERIT, and these increases are significant (LMM: $F_{3,8} = 53.74, P < 0.0001$).

In PERFECT-MERIT, there is zero meritocratic unfairness. Indeed, no bias in corrections that could reduce contributing behavior are predicted. Not even full-contributors who are matched in the lowest group due to bad luck decrease their contribution in the next round. In fact, this happened only 3 times in 210 occurrences of such bad luck. This striking result is as predicted. In fact, experiences of meritocratic unfairness are not possible under PERFECT-MERIT, and so participants do not perceive being placed in a lower group due to bad luck as unfair; it is part of the mechanism, and many subjects successfully manage to take turns in those unlucky positions.

In conditions HIGH, LOW, and NO-MERIT, we studied how the level of meritocratic unfairness experienced in the previous round impacts the decision to contribute in the following round. To do so, we performed a multilevel regression of between-rounds contribution adjustments with subject and session as random effects. Our findings reveal that disadvantageous unfairness leads to decreases in treatments LOW-MERIT $$-0.18^{***} (0.05)$$, and NO-MERIT $$-0.25^{***} (0.03)$$). For HIGH-MERIT the decrease is consistent in sign and size, but not statistically significant $$-0.39 (0.21)$$. However, if HIGH-MERIT and LOW-MERIT are pooled together the effect turns out to be significant $$-0.25^{***} (0.03)$$. Meritocratic disadvantageous fairness can, therefore, originate significant differences between the theoretical equilibrium predictions and experimentally observed behavior. Advantageous unfairness leads to increases under some but not under all regimes. The strengths of these effects varied and the evidence was not contrary to predictions.

We also performed additional regressions to compare meritocratic fairness to a standard notion of fairness, which we chose to be represented by Fehr and Schmidt (1999). As expected, applying the standard notion of distributional fairness yielded good results only for the case of NO-MERIT (for which it was conceived). On the other hand, meritocratic unfairness proved a good predictor of the contribution adjustments between rounds across all other treatments. Standard fairness did not prove to be a good predictor of contributions in these treatments, and decreasingly so for higher levels of meritocracy. Therefore, meritocratic fairness can be seen as a natural extension of distributional fairness in games with positive levels of meritocracy. Additional details and full regression tables are available in the Appendix. We leave it as an avenue for future work to analyze alternative fairness measures too, and to develop a general theory of fairness (which fairness consideration matters when?).

### Sensitivity

So far, we have shown that (i) both efficiency *and* equality increase with meritocracy, and that (ii) considerations of ‘meritocratic fairness’ may explain deviations from equilibrium predictions. In this section, we show that changes in the level of experienced meritocracy have significant implications as well. In particular, we test whether participants coming from a higher (lower) meritocracy level in part 1 are more (less) sensitive to meritocratic unfairness in part 2.

For this analysis, we used the data pertaining of part 2 of the experiment, controlling for which meritocracy level was played in part 1. We divided the dataset in two subsets, depending on whether participants in part 2 experienced a higher or lower meritocracy level than in part 1. In order to obtain a balanced design with respect to the direction of meritocracy changes, we further sampled the data from part 2 to include only the intermediate regimes of meritocracy ($\sigma ^2=\{3,20\}$). In this way, both conditions could be tested against perfect meritocracy, zero meritocracy, and one intermediate regime. We created a dummy variable for “contribution goes down” (0;1) and performed a multilevel logistic regression with subject and session as random effects. We used the level of disadvantageous meritocratic unfairness experienced in the previous round as a predictor of whether contribution is expected to go up or down in the next round.

Our main finding is that the distaste for meritocratic unfairness is exacerbated after having played a more meritocratic regime in part 1. That is, if a participant experienced meritocratic unfairness in the previous round, he/she is more likely to reduce the own contribution in the current round if the level of meritocracy in part 2 is lower than in part 1 (Logistic Mixed Regression LMR: $Z = 2.521, P = 0.0117$). The effect in the opposite direction—a lower meritocracy level in part 1 than in part 2—is not significant (LMR: $Z = 1.522, P = 0.128$).

The different sensitivity to meritocratic unfairness may explain the different levels of efficiency and equality overall. Sessions in part 2 with higher sensitivity to meritocratic unfairness—i.e. descending the meritocracy ladder—have significantly lower average payoff (One-sided Kolmogorov-Smirnoff KS: $D^+ = 0.1531, P < 0.0001$), and significantly higher inequality—measured by the average Gini coefficient per round ($D^+ = 0.1583, P = 0.0494$). These results confirm that, in our settings, increases in efficiency are followed by inequality reduction, and that meritocratic fairness considerations may help explain this dissolution of the predicted efficiency-equality tradeoff.

## Discussion

Our aim was to investigate the welfare consequences of institutions implementing a fuzzy mechanism instead of a perfect one. We initiated this line of research by considering a novel mechanism where contributors have a tendency to be matched with contributors, and free-riders with free-riders. Under such a mechanism, it was predicted that zero meritocracy leads to maximal equality and minimal efficiency, while perfect meritocracy to the opposite. Regarding fuzzy implementations of meritocracy, theory predicted ‘leaky buckets’ in both directions: reducing meritocracy increases equality at the expense of efficiency, and increasing meritocracy increases efficiency at the expense of equality. These predictions reflect a tradeoff that is at the core of economic theory (Arrow 1951; Sen 1970; Okun 1975; Gauthier 1986; Arrow et al. 2000).

In our study, we analyzed the efficiency-equality tradeoff in a controlled laboratory experiment. We explored a range of intermediate ‘fuzzy’ meritocracy regimes motivated by the fact that real-world mechanisms would typically not be perfectly precise. We consider a high-fuzziness implementation the default for a real-world implementation, but consider the possibility that a policy maker could make investments to reduce the noise of the monitoring/implementation. When we consequently interpret setting the level of precision of the mechanism—for the moment without considering the costs that such an activity would entail—as a policy choice, we obtained the following result. Surprisingly, we found that the tradeoff is dissolved behaviorally. Matching mechanisms that are more meritocratic and that, therefore, promise higher efficiency from a theoretic point of view, also turn out to benefit the worst-off and to improve overall distributional equality. Theory predicted otherwise. This result was true even in parameter ranges where the high-efficiency equilibria did not exist. This suggests that any fuzzy version of meritocratic matching would be beneficial to implement.

Our results rely on two critical assumptions. First, our experiments feature ex ante equity. Indeed, this is an important prerequisite, as meritocratic matching generally does not enable high-efficiency equilibria with heterogeneity amongst agents (Duca et al. 2016). Second, group sizes are fixed. While alternative models have been proposed (Cinyabuguma et al. 2005; Ehrhart and Keser 1999; Ahn et al. 2008; Coricelli et al. 2004; Page et al. 2005; Brekke et al. 2007; Brekke 2011; Charness and Yang 2014), exploring combinations of endogenous group-formation and meritocratic matching is left as an avenue for future research.

## Figures and Tables

**Fig. 6:**
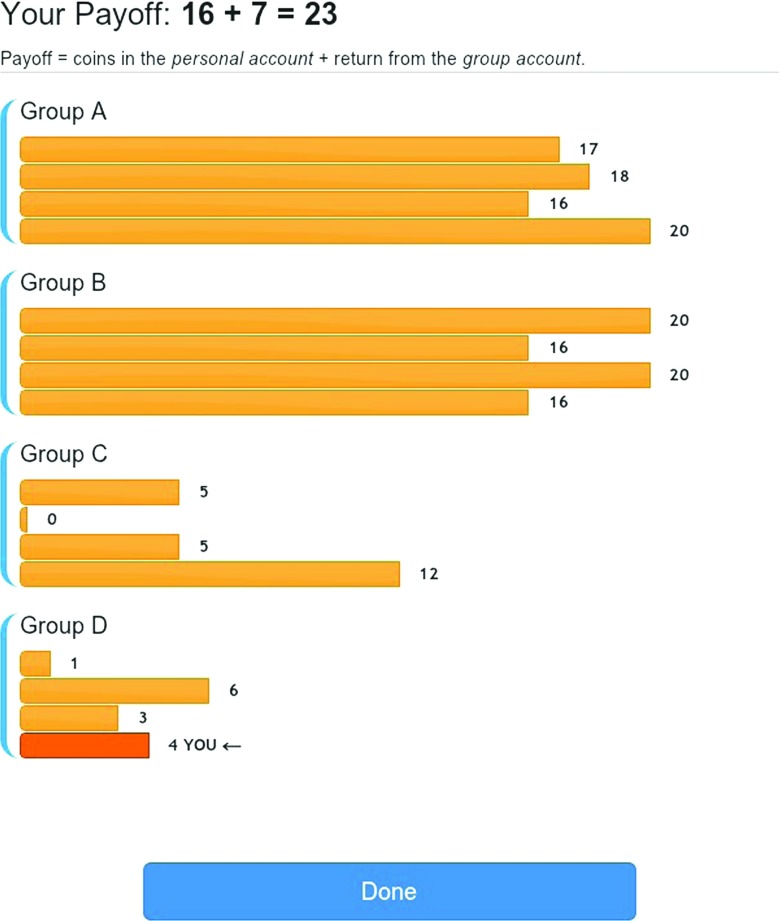
Game interface for displaying the results. Participants’ contribution decisions are displayed as horizontal bars of variable length sorted according to their ranking after noise has been applied

**Fig. 7:**
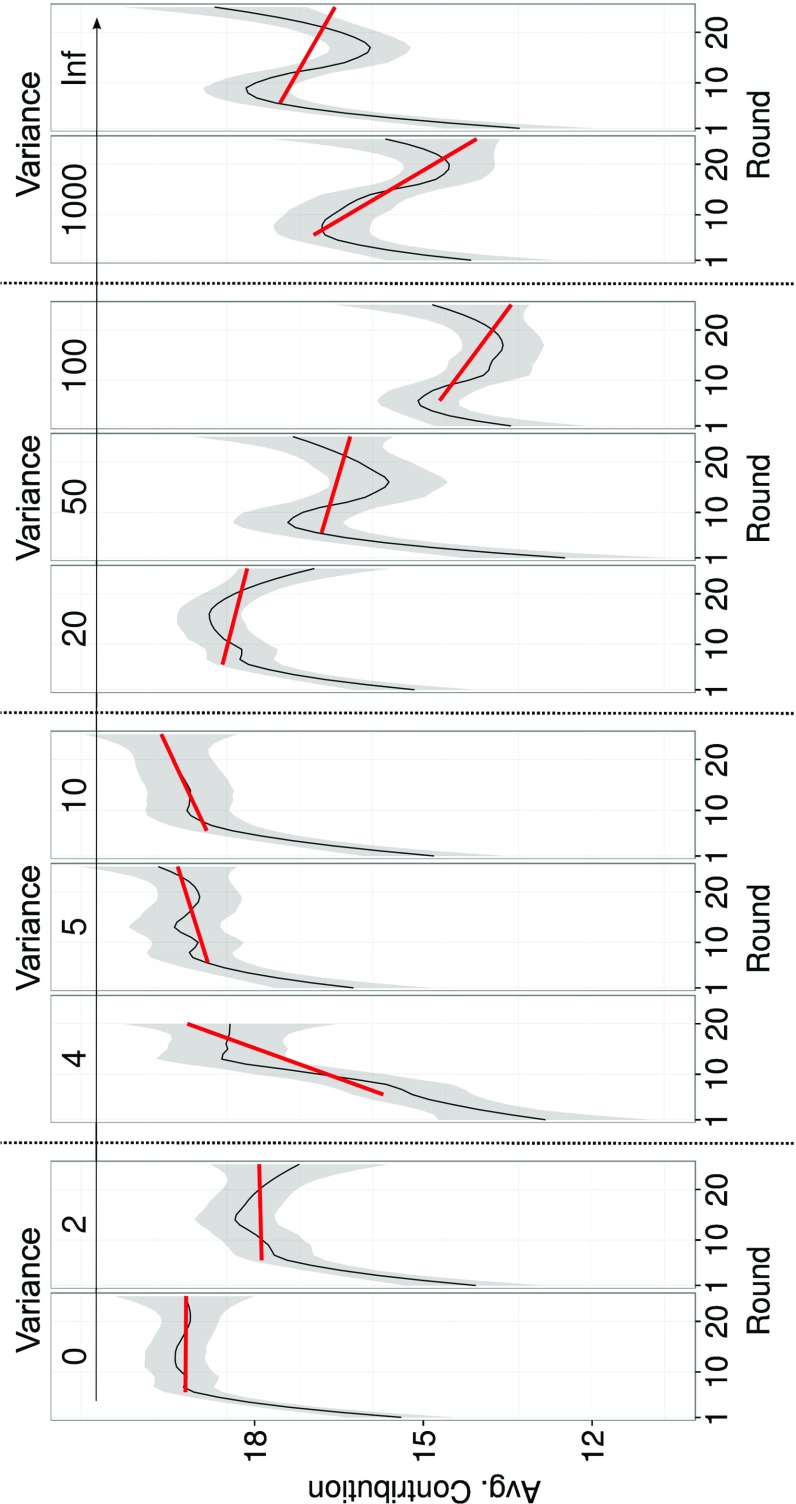
Average contribution levels over time for different levels of variance in experiments played online. Approximately four contribution regimes were found: (i) from 0 to 2 players’ contributions stabilize immediately; (ii) from 4 to 10 players’ contributions are increasing tending towards the high-efficiency Nash equilibrium; (iii) from 20 to 50 players’ contributions are declining towards the zero-efficiency equilibrium; (iv) for extremely high-levels from 1000 to Infinity, the decline of players’ contributions is even steeper. The red line shows a fitted linear regressions on the data excluding the first five rounds where players are still learning the dynamics of the game

**Fig. 8:**
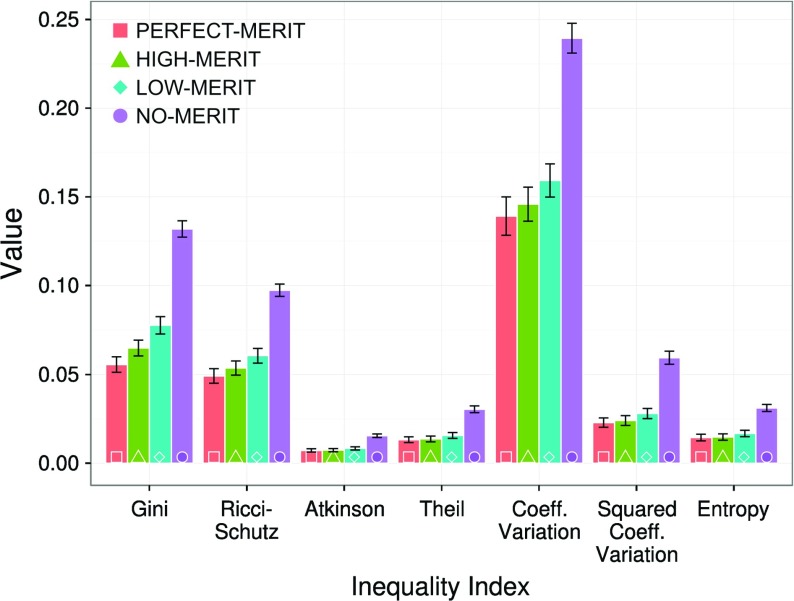
Battery of indexes measuring payoff inequality over the forty rounds for perfect-, high-, low-, and no-meritocracy, respectively associated with the values of $\sigma ^2=\{0,3,20,\infty \}$. Inequality decreases with meritocracy for a large number of distinct inequality indexes. Error bars represent the 95%-confidence intervals

## A Materials and methods

### A.1 Equilibrium structure

Our stage games with $n=16$, $s=4$, $B=20$ and $m=0.5$ have the following equilibria dependent on which variance level of $\sigma ^2=\{0,3,20,\infty \}$ is played. When $\sigma ^2=\infty$ (NO-MERIT), the only equilibrium is $c_i=0$ for all *i*. $c_i=0$ for all *i* is also an equilibrium for all other variance levels. In that equilibrium, all players receive a payoff of $\phi _i=20$. However, when $\sigma ^2=\{0,3,20\}$, there also exist exactly ${n\atopwithdelims ()k}$ unique pure-strategy equilibria such that $c_i=0$ for exactly two agents and $c_j=20$ for the remaining fourteen. In that equilibrium, for the case when $\sigma ^2=0$ (PERFECT-MERIT), payoffs are such that twelve of the fourteen players who contribute $c_i=20$ are matched in groups with each other and receive $\phi _i=40$. The remaining four players are matched in the worst group. Of those, the two players who contribute $c_i=0$ receive a payoff of $\phi _i=40$, while the two players who contribute $c_i=20$ receive a payoff of $\phi _i=20$. For the cases when $\sigma ^2=3$ (HIGH-MERIT) and $\sigma ^2=20$ (LOW-MERIT), payoffs in the last group are as in the case when $\sigma ^2=0$ (PERFECT-MERIT) in vast majority of all cases ($\ge 95\%$). In the remaining cases, payoffs are such that out of fourteen players who contribute $c_i=20$ are matched in groups with each other and receive $\phi _i=40$. The remaining 6 players who contribute $c_i=20$ are matched in a group with one player who contributes $c_i=0$ and receives a payoff of 30. The two players who contribute $c_i=0$ receive a payoff of $\phi _i=50$ each. Analysis of the results of ten thousands computer simulations performed in MATLAB showed that the near-efficient Nash equilibrium collapses when the variance reaches a level of about $\sigma ^2=75$ (see propositions 6 and 7 in Ref. Nax et al. 2014).

### A.2 Experimental design of the lab experiment

A total of 192 voluntary participants took part in one session consisting of two separate games each. Each session lasted roughly one hour. There were 16 participants in each session and 12 sessions in total. All sessions were conducted at the ETH Decision Science Laboratory (DeSciL) in Zürich, Switzerland, using the experimental software NodeGame (Balietti 2016). DeSciL recruited the subjects from the joint subject pool of the University of Zurich and ETH Zurich maintained by the University Registration Center for Study Participants (UAST).^19^ The experiment followed all standard behavioral economics procedures and meets the ethical committee guidelines. Decisions, earnings and payments were anonymous. Payments were administered by the DeSciL administrators. In addition to a 10 CHF show-up fee, each subject was paid according to a known exchange rate of 0.01 CHF per coin. Overall, monetary rewards ranged from 30 to 50 CHF, with a mean of 39 CHF.

Each session consisted of two games, each of which was a forty-round repetition of the same underlying stage game, namely a public-goods game. The same fixed budget was given to each subject every period. Each game had separate instructions that were distributed at the beginning of each game. After reading the instructions, all participants were quizzed to make sure they understood the task. The two games differ with respect to the variance level that is added to players’ contributions. There were four variance levels ($\sigma ^2=\{0,3,20, \infty \}$), and each game had equivalent instructions. Instructions contained full information about the structure of the game and about the payoff consequences to themselves and to the other agents. We played every possible pair of variance levels in both orders to have an orthogonal balanced design, which yields a total of 12 sessions. As the game went on, players learnt about the other players’ previous actions and about the groups that formed. Each of our 192 participants made forty contribution decisions in each of the two games in his session. This yields 80 choices per person per session, hence a total of 15,360 observations. More details, including a copy of a full instructions set, are provided in the following subsections.

#### Instructions of the lab experiment

Each experimental session consisted of two separate games (part 1, part 2), each played with a different variance level. We exhausted all possible pair of variance levels in both orders, for a total of 12 different combinations. Consequently, we prepared 12 different instruction texts that took into account whether a variance level was played in the first or in the second part, and in the latter case also considered which variance level was played in part 1.

Together with the main instructions sheet, we provided an additional sheet containing tabulated numerical examples of fictitious game-rounds played at the current variance level. This aimed to let participants get an intuitive feeling of the consequences of noise on contributions and final payoffs.

All instructions texts can viewed at the address http://nodegame.org/games/merit/. Here we report the instruction text for variance level equal 20 played in the part 1.

#### Instructions for variance level = 20, part 1

Welcome to the experiment and thanks for your participation. You have been randomly assigned to an experimental condition with 16 people in total. In other words you and 15 others will be interacting via the computer network for this entire experimental session.

The experiment is divided into two parts and each part will last approximately 30–40 min long. Both parts of the experiment contribute to your final earnings. The instructions for the first part of the experiment follow directly below. The instructions for the second part of the experiment will be handed out to you only after all participants have completed the first part of the experiment. It is worth your effort to read and understand these instructions well. You will be paid based on your performance in this study; the better you perform, the higher your expected earnings will be for your participation today.

**Your decision.** In this part you will play 40 independent rounds. At the beginning of each round, you will receive 20 “coins”. For each round, you will have to decide how many of your 20 coins to transfer into your “personal” account, and how many coins to transfer into a “group” account. Your earnings for the round depend on how you and the other participants decide to divide the coins you have received between the two accounts.

**Group matching with noise.** For each round you will be assigned to a group of 4 people, that is, you and three other participants. In general, groups are formed by ranking each individual transfer to the group account, from the highest to the lowest. Group 1 is generally composed of those participants who transferred the most to the group account; Group 4 is generally composed of those who transferred the least to the group account. The other groups (2 and 3) are between these two extremes.

However, the sorting process is noisy by design; contributing more will increase a participant’s chances of being in a higher ranked group, but a high ranking is not guaranteed. Technical note- The noisy ranking and sorting is implemented with the following process:

- ***Step 1:*** Preliminary ordering. A preliminary list is created in which transfers to the group account are ranked from highest to lowest. In case two or more individuals transfer the same amount, their relative position in the ranking will be decided randomly.
- ***Step 2:*** Noisy ordering. From every participant’s actual transfer to the group account, we obtain a unique noisy contribution by adding an i.i.d. (independent and identically distributed) normal variable with mean 0 and variance 20. The noisy contributions are then ranked from 1 to 16 from highest to lowest, and a final list is created.
- ***Step 3:*** Group matching. Based on the final list created at Step 2 (the list with noise), the first 4 participants on that list form Group 1, the next 4 people in the list form Group 2, the third 4 people in the list form Group 3, and the last 4 people form Group 4.

**Return from personal account.** Each coin that you put into your personal account results in a simple one-to-one payoff towards your total earnings.

**Return from group account.** Each coin that you put into the group account will pay you back some positive amount of money, but it depends also on how much the other group members have transferred to the group account, as described below.

The total amount of coins in your group account is equal to the sum of the transfers to the group account by each of the group members. That amount is then multiplied by 2 and distributed equally among the 4 group members. In other words, you will get a return equal to half of the group account total.

**Final earnings.** Your total earnings for the first part of the experiment are equal to the sum of all your rounds’ earnings. One coin is equal to 0.01 CHF. This may not appear to be very much money, but remember there are 40 rounds in this part of the experiment so these earnings build up.

**Example.** Here is an example of one round to demonstrate this decision context, the noisy sorting into different groups, and the different resulting payoffs. In the table below, pay attention to the following facts:

- Groups are roughly formed by ranking how much participants transferred to the group account, but this is not a perfect ranking. For example, participant #8 transferred less to the group account than participant #10, but the noisy sorting process placed him in a higher ranked group.
- Participant #7 transferred 14 of his coins to the group account. This means that he transferred 6 to his personal account. Due to noisy sorting he was ranked first, and assigned to Group 1. The other participants in Group 1 transferred a total of 64 coins to the group account. This amount is doubled and redistributed evenly back to the 4 members of the group this is 32 for each participant. So then participant #7 earned 38 coins for this round.
- Participant #12 transferred 7 coins to the group account and transferred the remaining 13 coins to his personal account. He was sorted (with noise) into Group 3 and this group transferred 46 coins in total. This resulted in 23 coins being returned to each of the group members, and thus his total payoff is 36 coins (23 returned from the group account and the 13 he kept in his personal account).

**Table:**

Player ID	Group	Transfer to group account	Transfer to personal account	Total to group account	Amount returned to player	Total earnings for the round
7	1	14	6	64	32	38
6	1	13	7	64	32	39
14	1	16	4	64	32	36
4	1	8	12	64	32	44
1	2	14	6	51	25.5	31.5
3	2	20	0	51	25.5	25.5
8	2	11	9	51	25.5	34.5
11	2	19	1	51	25.5	26.5
10	3	17	3	46	23	26
12	3	7	13	46	23	36
16	3	6	14	46	23	37
5	3	16	4	46	23	27
9	4	10	10	18	9	19
2	4	1	19	18	9	28
13	4	5	15	18	9	24
15	4	2	18	18	9	27

Additional examples are provided in a separate sheet for your own reference.

#### Quiz

Subjects were given a quiz after instructions to test their understanding of the game. Only after “passing” the quiz were subjects allowed to begin play. Details about the quiz can be found at http://nodegame.org/games/merit/.

#### Graphical interface of the experiment

The experiment was implemented using the experimental software nodeGame (Balietti 2016). Besides, offering a textual response of the actions of the players, we also offer a visual summary with contributions bars ordered by group, as shown in Fig. 6. More details about the interface, and the implementation are available at the url: http://nodegame.org/games/merit/.

### A.3 Statistical analyses

#### Online sessions

Table 2 offers an overview of the 16 online experimental sessions performed on Amazon Mechanical Turk (AMT). Sessions lasted 20–25 rounds and, due to dropouts (Arechar et al. 2017), some sessions have less observations than the expected, i.e. number of players (16) times number of rounds.

**Table 2:** Descriptive statistics for online sessions on Amazon Mechanical Turk

Id	Variance	Rounds	Obs	Contribution	SD	SE	CI
1	0	20	300	19.03	3.21	0.19	0.36
2	0	25	398	17.95	5.43	0.27	0.54
3	2	20	300	16.73	5.91	0.34	0.67
4	2	25	400	16.85	6.03	0.30	0.59
5	4	20	320	15.86	6.40	0.36	0.70
6	5	25	400	18.45	4.91	0.25	0.48
7	10	25	331	18.19	4.60	0.25	0.50
8	20	20	280	18.70	3.74	0.22	0.44
9	20	25	397	16.64	5.62	0.28	0.55
10	50	25	400	15.35	6.33	0.32	0.62
11	100	20	240	13.08	6.37	0.41	0.81
12	100	25	379	13.01	5.74	0.30	0.58
13	1000	20	320	14.06	7.95	0.44	0.87
14	1000	25	351	15.35	6.08	0.32	0.64
15	$\infty$	20	240	14.60	6.73	0.43	0.86
16	$\infty$	25	400	16.79	5.63	0.28	0.55

We performed multilevel regressions on contributions over time with subject and session as random effects. The results can be summarized as follows:

- $\sigma ^2=[0\ldots 50]$: contributions are significantly increasing;
- $\sigma ^2=100$: contributions are not significantly decreasing;
- $\sigma ^2=1000$: contributions are significantly decreasing;
- $\sigma ^2=\infty$: contributions are not significantly increasing.

All contribution trends are as expected, except for $\sigma ^2=\infty$. We interpreted this surprising result as a random fluctuation within the small sample collected. In fact, it is known that online experiments tend to give noisier results than laboratory ones (Gaudecker et al. 2012). As a partial confirmation of our interpretation, the trend is reversed if we exclude the first 5 rounds—assuming that some sort of group learning is going on. Figure 7 shows the contribution levels over time aggregated by variance level.

#### Equality analysis—Gini coefficients

The Gini index differs significantly among the four treatments (LMM: $F_{3,20} = 42.0, P < 0.0001$). Taking NO-MERIT as a baseline, LOW-MERIT led to a decrease in the variance of realized payoff in each round of −0.058901 (LRT $\chi _{(1)} = 18.18, P < 0.0001$), HIGH-MERIT to a decrease of −0.071843 (LRT $\chi _{(1)} = 22.28, P < 0.0001$), and PERFECT-MERIT to a decrease of $-0.075453$ (LRT $\chi _{(1)} = 22.06, P<0.0001$). Computing Bonferroni adjusted *p*-values for all pair-wise differences reveals that the treatment with variance $\infty$ is significantly different ($P < 0.0001$) from the other three variance levels ($\sigma ^2=\{0,3,20\}$), which are themselves not significantly different from each other (see Fig. 4 in the main text).

#### Fairness analysis

We find a significant difference in the experienced levels of meritocratic unfairness in each round among the four treatments (LMM: $F_{3,8} = 53.74, P < 0.0001$). When computing Bonferroni adjusted *p*-values we find that—excluding PERFECT-MERIT for which meritocratic unfairness is always zero by definition—all treatments are statistically significantly different from each other (HIGH vs LOW-MERIT $P = 0.0071$, all the other pair-wise comparisons $P < 0.0001$). Taking NO-MERIT as a baseline, LOW-MERIT led to a decrease in the experienced meritocratic unfairness in each round of −1.66 (LRT $\chi _{(1)} = 11.76, P = 0.0006$), HIGH-MERIT to a decrease of −2.36 (LRT $\chi _{(1)} = 18.92, P < 0.0001$).

#### Fairness regressions

Here we report the results of the mixed-effects regressions of meritocratic and distributional fairness on contributions adjustments between rounds in part 1 and part 2 of the experiment. As we argued in the main text, distributional fairness cannot be easily generalized to the case of assortative matching. Here we show that a näive extension of the formula in Fehr and Schmidt (1999) fails to reproduce the results predicted by theory. In fact, both within-group and across-groups distributional fairness under assortativity often lead to the contradictory result that disadvantageous fairness implies an increase in the contribution levels. However, by taking into account assortativity in the formula of distributional fairness, we developed an extension that is able to reproduce the results predicted by the theory for all treatments.

#### Meritocratic fairness

In Tables 3 and 4, meritocratic unfairness is used as a predictor. lag.merit.fair.dis and lag.merit.fair.adv are respectively the amount of *disadvantageous* and *advantageous* meritocratic unfairness experienced by a player in the previous round, measured according to the equations in Sect. 2 of the main text.

**Table 3:** Meritocratic fairness predicts contribution differential. (Part 1)

	HIGH-MERIT	LOW-MERIT	HIGH-MERIT&LOW-MERIT	NO-MERIT
(Intercept)	0.25	0.15	0.03	0.03
	(0.16)	(0.16)	(0.19)	(0.19)
lag.merit.fair.dis	$$-0.39$$	$$-0.18^{***}$$	$$-0.25^{***}$$	$$-0.25^{***}$$
	(0.21)	(0.05)	(0.03)	(0.03)
lag.merit.fair.adv	$$-0.91^{**}$$	0.06	$$0.15^{***}$$	$$0.15^{***}$$
	(0.30)	(0.06)	(0.03)	(0.03)
AIC	12,314.36	12,284.05	12,359.50	12,359.50
BIC	12,347.56	12,317.24	12,392.70	12,392.70
Log likelihood	−6151.18	−6136.02	−6173.75	−6173.75
Num. obs.	1872	1870	1872	1872

*** $$p<0.001$$, ** $$p<0.01$$, * $$p<0.05$$

The sign of the regression coefficient is always consistent with theory predictions. HIGH-MERIT is significant if pooled together with LOW-MERIT

**Table 4:** Meritocratic fairness predicts contribution differential. (Part 2)

	HIGH-MERIT	LOW-MERIT	HIGH-MERIT&LOW-MERIT	NO-MERIT
(Intercept)	0.13	0.16	0.11	$0.38^{*}$
	(0.16)	(0.17)	(0.11)	(0.18)
lag.merit.fair.dis	$$-0.45$$	$$-0.29^{***}$$	$$-0.29^{***}$$	$$-0.26^{***}$$
	(0.28)	(0.07)	(0.06)	(0.02)
lag.merit.fair.adv	$-0.57$	0.00	$-0.02$	0.04
	(0.32)	(0.07)	(0.07)	(0.02)
AIC	12,288.63	12,419.05	24,699.24	12,123.03
BIC	12,321.83	12,452.25	24,736.60	12,156.23
Log Likelihood	−6138.31	$-6203.53$	−12,343.62	−6055.51
Num. obs.	1872	1871	3743	1872

*** $$p<0.001$$, ** $$p<0.01$$, * $$p<0.05$$

The sign of the regression coefficient is always consistent with theory predictions. HIGH-MERIT is significant if pooled together with LOW-MERIT

**Table 5:** Within-group distributional fairness predicts contribution differential. (Part 1)

	PERFECT-MERIT	HIGH-MERIT	LOW-MERIT	HIGH-MERIT & LOW-MERIT	NO-MERIT
(Intercept)	$$-0.79^{***}$$	$$-1.39^{***}$$	$$-1.32^{***}$$	$$-1.39^{***}$$	$$1.40^{**}$$
	(0.23)	(0.22)	(0.21)	(0.15)	(0.45)
lag.distr.fair.group.dis	$$-0.03$$	$$0.13^{**}$$	0.01	$$0.06^{*}$$	$$-0.70^{***}$$
	(0.04)	(0.05)	(0.05)	(0.03)	(0.04)
lag.distr.fair.group.adv	$$0.76^{***}$$	$$0.99^{***}$$	$$0.77^{***}$$	$$0.88^{***}$$	$$0.28^{***}$$
	(0.04)	(0.04)	(0.04)	(0.03)	(0.04)
AIC	11,682.40	11,933.18	12,025.27	23,952.86	11,968.23
BIC	11,715.59	11,966.38	12,058.46	23,990.22	12,001.43
Log likelihood	−5835.20	−5960.59	−6006.64	−11,970.43	−5978.12
Num. obs.	1872	1872	1870	3742	1872

*** $$p<0.001$$, ** $$p<0.01$$, * $$p<0.05$$

The sign of the regression coefficient is often inconsistent with theory predictions

**Table 6:** Within-group distributional fairness predicts contribution differential. (Part 2)

	PERFECT-MERIT	HIGH-MERIT	LOW-MERIT	HIGH-MERIT & LOW-MERIT	NO-MERIT
(Intercept)	$$-0.93^{***}$$	$$-1.54^{***}$$	$$-1.25^{***}$$	$$-1.43^{***}$$	$$1.60^{***}$$
	(0.25)	(0.40)	(0.23)	(0.22)	(0.38)
lag.distr.fair.group.dis	$$-0.10^{*}$$	0.05	$$-0.06$$	0.00	$$-0.61^{***}$$
	(0.04)	(0.04)	(0.05)	(0.03)	(0.03)
lag.distr.fair.group.adv	$$0.88^{***}$$	$$1.19^{***}$$	$$0.86^{***}$$	$$1.02^{***}$$	$$0.15^{***}$$
	(0.04)	(0.04)	(0.04)	(0.03)	(0.03)
AIC	11,856.01	11,799.36	12,109.33	23,935.12	11,827.92
BIC	11,889.21	11,832.55	12,142.53	23,972.48	11,861.12
Log likelihood	−5922.01	−5893.68	−6048.67	−11,961.56	−5907.96
Num. obs.	1871	1872	1871	3743	1872

*** $$p<0.001$$, ** $$p<0.01$$, * $$p<0.05$$

The sign of the regression coefficient is often inconsistent with theory predictions

**Table 7:** Across-group distributional fairness predicts contribution differential. (Part 1)

	PERFECT-MERIT	HIGH-MERIT	LOW-MERIT	HIGH-MERIT & LOW-MERIT	NO-MERIT
(Intercept)	$$-1.42^{***}$$	$$-2.40^{***}$$	$$-2.20^{***}$$	$$-2.23^{***}$$	$$1.04^{*}$$
	(0.26)	(0.34)	(0.34)	(0.24)	(0.40)
lag.distr.fair.dis	$$0.22^{***}$$	$$0.39^{***}$$	$$0.33^{***}$$	$$0.35^{***}$$	$$-0.44^{***}$$
	(0.03)	(0.04)	(0.04)	(0.03)	(0.05)
lag.distr.fair.adv	$$0.44^{***}$$	$$0.59^{***}$$	$$0.43^{***}$$	$$0.48^{***}$$	$$0.13^{*}$$
	(0.08)	(0.10)	(0.08)	(0.06)	(0.05)
AIC	11,934.03	12,223.59	12,225.86	24,434.15	12,277.90
BIC	11,967.23	12,256.79	12,259.05	24,471.51	12,311.10
Log likelihood	−5961.02	−6105.80	−6106.93	−12,211.07	−6132.95
Num. obs.	1872	1872	1870	3742	1872

*** $$p<0.001$$, ** $$p<0.01$$, * $$p<0.05$$

The sign of the regression coefficient is often inconsistent with theory predictions

**Table 8:** Across-group distributional fairness predicts contribution differential. (Part 2)

	PERFECT-MERIT	HIGH-MERIT	LOW-MERIT	HIGH-MERIT & LOW-MERIT	NO-MERIT
(Intercept)	$$-2.15^{***}$$	$$-1.98^{***}$$	$$-2.19^{***}$$	$$-2.01^{***}$$	$$1.96^{***}$$
	(0.30)	(0.30)	(0.35)	(0.23)	(0.48)
lag.distr.fair.dis	$$0.21^{***}$$	$$0.29^{***}$$	$$0.30^{***}$$	$$0.29^{***}$$	$$-0.49^{***}$$
	(0.03)	(0.03)	(0.04)	(0.02)	(0.04)
lag.distr.fair.adv	$$0.65^{***}$$	$$0.54^{***}$$	$$0.46^{***}$$	$$0.48^{***}$$	$$-0.04$$
	(0.09)	(0.09)	(0.09)	(0.06)	(0.04)
AIC	12,162.64	12,222.36	12,374.95	24,584.87	12,068.03
BIC	12,195.83	12,255.56	12,408.15	24,622.23	12,101.23
Log likelihood	−6075.32	−6105.18	−6181.48	−12,286.43	−6028.02
Num. obs.	1871	1872	1871	3743	1872

***$$p<0.001$$, **$$p<0.01$$, *$$p<0.05$$

The sign of the regression coefficient is often inconsistent with theory predictions

#### Distributional fairness

The results of the regressions for distributional fairness are shown in Tables 5, 6, 7 and 8. Based on the original formula in Ref. Fehr and Schmidt (1999), we tried two different extensions of the notion of distributional fairness for meritocratic environments. First, we computed distributional fairness for each player only taking into account the other players within the group into which he/she was matched (Within-group distributional fairness). The regressors in this case are called: lag.distr.fair.group.dis and lag.distr.fair.group.adv. Then, we also computed distributional fairness across all players, regardless of the group they belonged to (Across-group distributional fairness). The regressors for across-group distributional fairness are called: lag.distr.fair.dis and lag.distr.fair.adv.

### A.4 Additional inequality indexes

As stated in the main text, inequality decreases as meritocracy increases. In this section, we show that our finding is robust to the type of inequality measurement chosen. Figure 8 displays the payoff inequality as measured by a number of different indexes commonly found in the literature of inequality studies (Atkinson 1970).

## Footnotes

1 Importantly, we use the terminology of ‘fuzzy mechanism’ and ‘fuzzy institution’ to refer to the imprecision of the mechanism, not about mechanism design with ‘fuzzy preferences’. Fuzzy mechanisms in our setting can range from perfectly precise to completely imprecise and random. This is different to the use of the word for example in ‘fuzzy social choice where standard social choice theory based on ‘crisp preferences is extended to the case of ‘fuzzy preferences’ that is, imprecise preferences, e.g. Blin and Whinston (1973) and Dutta (1987).

2 See also other applications such as oligopoly competition with noisy demand schedules (Green and Porter 1984), or self-enforced agency contracts (Levin 2003).

3 See, for example, Ambrus and Greiner (2012) for an investigation of imperfect monitoring and costly punishment, or Aoyagi and Fréchette (2009) for an experimental study of noisy iterated prisoner’s dilemmas.

4 See also Isaac et al. (1985) for early, alternative implementations.

5 The list of mechanisms is long; see Chaudhuri (2011) for a review, punishment (Fehr and Gächter 2000) being a particularly well-known candidate.

6 Ties are randomly broken.

7 See Gunnthorsdottir (2010) Theorem 1 for the theorem by which to compute these high-efficiency equilibria.

8
Nax et al. (2014) is a separate theory paper that develops the relevant predictions that are being tested here.

9 Note that, due to the i.i.d. random draws, the exact same two numbers are generated with probability zero, so that tie-breaking is only needed when $\sigma = 0$.

10 Higher contribution levels were observed online than in the lab for the $\sigma ^2=\infty$ condition. More details are available in the Appendix.

11 At $\sigma ^2=3$ the system became more volatile, and at $\sigma ^2=20$ the system became unstable.

12 See Theorem 1 in Gunnthorsdottir (2010) and Propositions 6 and 7 in Nax et al. (2014) for detailed proofs and game-theoretic characterization of these equilibria.

13 Further details on our computations to obtain equilibrium conditions for our parameter values can be found in Appendix A.1.

14 In order to run two variance levels per person, we play half of the 80 rounds of Gunnthorsdottir (2010) each time.

15 Kruskal–Wallis rank-sum test $\chi _{(1)}= 4.35, p = 0.0369$. Unit of observation is one session ($n1=n2=3$).

16 Ex ante inequality for $\sigma ^2=0$, evaluated at expected payoffs in equilibrium, there are 2 free-riders earning 40 coins, and 14 full-contributors earning 37.1 coins. Ex post inequality for $\sigma ^2=0$, evaluated at realized payoffs in equilibrium, we have 2 free-riders earning 40 coins, 12 (lucky) full-contributors earning 40 coins, and 2 (unlucky) full-contributors earning 20.

17 Additional analysis of the Gini coefficient with more statistical tests can be found in Appendix A.3.

18 Note also that $MU_{Adv}$ ($MU_{Dis}$)is (dis)advantageous, because the players enjoys higher (lower) contributions from others than he would if meritocratic matching was implemented without flaw. Note that, because of this definition, meritocratic unfairness cannot arise under PERFECT-MERIT (when $\sigma ^2=0$).
